# Exploring access to support services for medical students: recommendations for enhancing wellbeing support

**DOI:** 10.1186/s12909-024-05492-1

**Published:** 2024-06-17

**Authors:** Aisha Ali Hawsawi, Neil Nixon, Emily Stewart, Elena Nixon

**Affiliations:** 1 Institute of Mental Health, Mental Health and Clinical Neurosciences, University of Nottingham, Nottingham, UK; 2 Dar Al-Hekma University, Jeddah, Saudi Arabia; 3 Nottinghamshire Healthcare NHS Foundation Trust, Nottingham, UK

**Keywords:** Medical student wellbeing, Support services, Barriers, Help-seeking

## Abstract

**Background:**

Medical students have reported facing unique challenges in their academic journey that can have a significant impact on their mental health and wellbeing; therefore, their access to support services and wellbeing resources has been deemed crucial for dealing effectively with the various challenges they tend to face. While previous research has highlighted certain barriers affecting medical students’ help-seeking and access to wellbeing support more generally, there is a pressing need for more in-depth research into the factors that may hinder or facilitate medical students’ acceptability and uptake of the wellbeing resources available to them within institutional contexts. The current study aims to explore students’ perceptions and utilization of wellbeing interventions and welfare resources within a medical school setting, as well as the factors influencing their help-seeking attitudes or behaviours. Additionally, it seeks to instigate medical students’ deeper reflections on potential enhancements that could be applied to wellbeing resources so that they are better suited to address their needs.

**Methods:**

This study employed a qualitative design, involving semi-structured interviews and a series of focus groups with medical students at the University of Nottingham (UK). Post-interview, focus groups were deemed necessary to gain deeper insights into emerging findings from the interviews regarding students’ views on wellbeing support services. Qualitative data from the interviews was subjected to thematic analysis while a hybrid thematic analytic approach was applied for the focus group data in order to allow for both pre-defined themes from the interviews and newly emerging patterns to be identified and analysed in a combined manner.

**Results:**

Twenty-five participants took part in the semi-structured interviews and twenty-two participants were recruited in a total of seven focus groups. Thematic analysis findings identified several key barriers to medical students’ accessing wellbeing resources, including difficulties in finding relevant information, lengthy processes and difficulties receiving prompt help in urgent situations, learning environment issues, confidentiality concerns, and stigma around mental health. Student suggestions for the enhancement of wellbeing provision were centered around proposed improvements in the format that the relevant information was presented and in the structure underlying the delivery of support services.

**Conclusion:**

The study findings shed light on multi-faceted factors contributing to medical students’ challenges in accessing support services; and provided a deeper understanding of medical students’ wellbeing needs through a consolidation of their recommendations for the implementation of practical steps to address these needs. These steps can potentially inform key medical education stakeholders so that they can actively and proactively foster more supportive environments that may help improve medical students’ help-seeking, as well as their acceptability and uptake of wellbeing services.

## Background

Within the ever-evolving context of medical education, there has been an increasingly recognised need for addressing more effectively the wellbeing issues identified widely in the medical student population [1, 2]. Previous literature has highlighted the persistent challenges faced by medical students, marked by a significant prevalence of psychological distress 74% [3], which has been also manifested in problems relating to depression, burnout, anxiety, and diminished mental quality of life [4, 5]; these rates exceed those observed in same-age peers [4, 5] raising concerns about the overall resilience and mental health of medical students and, consequently, the future healthcare workforce [6].

The literature has indicated a complex mix of factors associated with medical students’ psychological distress which tends to escalate during medical school, with the peak year varying based on the type of distress studied [7, 8]. A number of studies have attributed this distress to the demanding curriculum, rigorous clinical rotations, extensive placements, and the responsibility for patient care, all of which are factors that have been assumed to be creating a stressful learning environment [7, 9, 10]. Further studies have reported unique challenges beyond those of an academic nature per se; these have been shown to contribute to mental health concerns, including financial issues, relationship pressures and pre-diagnosis of medical or mental health issues [11, 12]. This interplay of academic, professional, and personal demands highlights the need for a comprehensive approach in addressing these challenges. On top of the usual struggles, the advent of the COVID-19 pandemic introduced unprecedented disruptions to medical education; shifting to online learning, uncertainty about clinical rotations, decreased motivation, and the extra stress stemming from the pandemic situation itself, such as health concerns and isolation, made things even more challenging for medical students [13]. Prior literature [14] highlighted the exacerbation of existing challenges and the emergence of new stressors since the onset of the pandemic, emphasizing the need for adaptive and responsive approaches to support medical students amidst rapidly evolving circumstances.

Even though a considerable number of medical students are assumed to be dealing with mental health issues, many of them don’t reach out for help. Researchers have identified compounding factors, such as fear of being seen as weak and stress normalization, that can create a complex web of challenges that can result in hindering access to essential support services for medical students [15]. Reinforcing these findings, the qualitative results of a study conducted in the United Kingdom indicated that students experience psychological distress in silence, even in the presence of support systems, due to barriers such as fear of stigma and self-awareness gaps; while, crucially, trusting relationships within the medical school environment emerged as a key factor driving students to access support [16]. The exploration of the dynamics of existing mental health services available within institutional settings reveals additional layers of complexity. While the literature has acknowledged the value of supportive staff and a student-centred support system [17], issues such as clarity about available services and waiting lists emerge as potential obstacles to receiving effective psychological support [18]. A scoping review of 33 articles identified cost as a primary systemic barrier for medical students globally, alongside conflicts of interest, confidentiality concerns, power imbalances, and fears about treatment appropriateness; the review highlighted the complexity of obstacles faced by medical students in seeking mental health support and underlined the importance of addressing these various barriers within the university context [19].

Realizing the urgency of the situation, a range of initiatives and interventions has emerged—ranging from extensive mental health programs [20] to wellness activities [21, 22] aimed at enhancing medical student wellbeing. Studies have shown that students’ perception of a supportive system in their educational institution is linked to positive outcomes, particularly lower stress levels and improved mental health [23, 24]. However, the effectiveness of interventions, whether therapy-based, such as cognitive behavioral therapy or mindfulness training, or academically driven, such as pass/fail grading and curricular changes [25, 26], warrants further investigation, particularly in relation to the impact and acceptability of such interventions on the psychological wellbeing of medical students. In response to the pressing need to comprehend the challenges medical students face in accessing mental health support, this research aims to explore the specific challenges medical students face in accessing mental health support and wellbeing services and to suggest ways in which this support provision can be improved from the students’ perspective. Considering the growing concerns about student mental health in medical education and the highlighted need for institutions to provide appropriate support resources to promote medical students’ psychological wellbeing [1], the objective of the present study is to explore particularly the obstacles to help seeking and to accessing support systems within medical education. Addressing such issues early in medical students’ careers would ultimately contribute to the wellbeing of future healthcare professionals who have notably been reported to lie within the occupational sectors that are at risk for poor wellbeing and mental health problems [27]. The study’s overarching research question is: How can wellbeing resources offered within the medical school/University settings help support the psychological wellbeing of medical students?

## Methods

### Study design

This study utilized a qualitative design consisting of semi-structured interviews, followed by a series of focus groups. The interview questions focused overarchingly on topics pertaining to how or whether wellbeing support was accessed, as well as any identified barriers and recommendations for improvement. The focus groups aimed to deepen insights from interviews on wellbeing support by exploring in more depth medical students’ challenges in accessing school support and wellbeing services, including the specifics of how, when, and in what ways issues might occur, while also instigating medical students’ suggestions for improvements to this wellbeing support. To comply with COVID-19 ethics regulations during the period that the study took place (March 2021-March 2023), the interviews and focus groups were conducted remotely using Microsoft Teams. The study was approved by the University of Nottingham Faculty of Medicine and Health Sciences Research Ethics Committee [ref no: FMHS 1561 − 1120 (DoPAP review ref 1561)]. All study procedures and aspects of data management adhered to the University of Nottingham’s code of research conduct and ethics policies, including the Data Protection Policy and Handling Restricted Data Policy, cited within the approved ethics protocols.

### Participants

Medical students aged 18 to 41 years attending either an undergraduate or graduate programme at the University of Nottingham were invited to participate in the study. Participants were recruited using a convenience sampling approach, through a study flyer that was circulated through University communication channels and student year email notifications. The researcher (AH) also reached out to student representatives, the student union and the director of student wellbeing for their help in disseminating the study flyer through the University-approved channels of communication. The focus groups could include participants who had participated in the interviews at an earlier stage. According to previous study protocols and practices [28, 29], the estimated approximate number of participants needed for either part of the study was 25, depending upon data saturation. Prospective participants were directed via the study flyer to follow a link to an online survey (Online Surveys) whereby they could access the Participant Information Sheet that outlined the study details, followed by a Consent Form. Participants were encouraged through the participant information sheet to contact the researcher with any questions they had prior to consenting. Upon signing the consent form, participants were directed next to a short online demographics form. After obtaining consent, participants were contacted by the researcher (AH) via email to schedule their participation in the interviews and/or focus groups, facilitated through MS Team invitations with allocated timeslots.

### Procedure

In collaboration with academic staff and students, the research team developed the semi-structured interview guide following an extensive literature review. One pilot interview was conducted to improve the flow and depth of the interview process and help refine the prompts to be used in the interviews. The semi-structured interview guide consisted of several broad, open questions followed by closed questions or probes to encourage more detailed or in-depth responses. Building upon insights from earlier interviews, the focus group guide was shaped utilizing pre-defined codes derived from the interviews. The focus-group guide aimed to foster cohesive and engaging discussions, incorporating relevant interview quotes tied to the identified codes.

Before the interview and the focus groups took place, participants were explicitly reminded that, as they were advised in the Participant Information Sheet, they were not obliged to answer any questions that would make them uncomfortable or that they chose not to address, and also that their participation would not impact their studies. For safeguarding purposes, links to support services were also provided should any participant experience distress during or after participating in the study. A £50 Amazon gift voucher was offered in a prize draw post-interviews/focus groups as an inconvenience allowance. The interviews lasted between 30 and 60 minutes, and the focus groups lasted 60 to 90 minutes. Data collected through the interview and focus groups were audio-recorded, transcribed verbatim, and saved anonymously on University-approved OneDrive folders for analysis, separately from participants’ consenting and demographic data.

### Interview data analysis

Thematic analysis of the study data was conducted using Braun & Clark’s [29–31] established six-step methodological approach, which enabled the identification of patterns and themes within the qualitative data. The themes were formulated based on the content of the data, allowing for an inductive thematic analysis approach to identify any themes that would emerge from the participants’ data. Semi-structured interviews were conducted by AH and transcribed by AH and a co-author (ES), with ES transcribing 70% of the material; AH and ES recorded initial thoughts to familiarise themselves with the data before creating preliminary codes, which were discussed with the research team, in particular co-authors EN and ES. One medical student reviewed the interview transcriptions, and AH incorporated all the suggested corrections. Codes were identified using the NVivo software (version 12), and a list of produced codes was reviewed by AH and ES until a consensus was reached. As the study progressed, a streamlined process was adopted, focusing on retaining core themes over minor themes that didn’t end up being representative of the majority of the participant sample. Codebooks were employed to maintain consistency in the thematic analysis processes, following recommended practice; additionally, a reflexive log was kept to document and analyze data transcriptions, coding, personal reflections, or observations that emerged during the analysis process [31, 32].

### Focus group data analysis

The focus group study utilized a hybrid thematic analysis, integrating both inductive and deductive approaches [33]; this combined method incorporated insights from interviews and validated previous research while also exploring the effective implementation of recommendations within existing support services. To ensure a systematic approach, the recorded focus group sessions were transcribed verbatim and organized in a spreadsheet; these transcriptions underwent a thorough review by AH, who made direct edits as necessary to maintain the anonymity of the data. The analysis used a dual-coding approach, incorporating into the focus-group guide a priori codes derived from the interviews; both a priori and posteriori codes were derived from the focus group transcripts, recorded separately for clarity [33]. The theming process focused on core themes, iteratively grouping codes until cohesive themes emerged. This approach aimed to capture the complexity of the data, incorporating deductive and inductive elements. The thematic findings from the interviews and focus groups were synthesized following standard guidelines for reporting qualitative research [34].

## Results

### Interview results

The demographics of the 25 participants are outlined in Table 1.

**Table 1:** Participant demographic characteristics

Demographics of participants	Number (*n*, %) Interview	Number (*n*, %) Focus group
Gender		
Female	21(84%)	18(82%)
Male	4 (16%)	4(18%)
Age in years		
18–21	12 (48%)	10 (45%)
22–25	9 (36%)	10 (45%)
26–29	2 (8%)	2 (10%)
30–33	2 (8%)	0 (%)
Year in school		
Pre-clinical	5 (20%)	5 (23%)
Clinical	20 (80%)	17 (77%)
Undergraduate	22 (88%)	20 (91%)
Graduate-entry students	3 (12%)	2 (9%)

The findings were organized into six primary themes, as outlined in Table 2, which highlighted the outcomes of the interview thematic analysis. These themes illuminated the barriers that medical students identified to accessing support services and students’ suggestions for potential avenues for improvement.

**Table 2:** Interview thematic analysis outcomes

Themes	Quotes
Theme 1 - Difficulty finding the wellbeing information prevent access to support	“It was difficult to find that information, and I don’t think it should be difficult to find that information” P7, Y4
Theme 2 - Long processes and waiting lists are deterring factors in help-seeking	“The waiting times can be pretty bad for the official university support counseling” P4, Y5
Theme 3 - Confidentiality and fear of negative judgment are barriers to seeking help	“Fitness to practice was a big thing, so I was really worried that if I made it known that I needed kind of extra help, I was worried that I would kind of be questioned about my ability to be a doctor” P8, Y3
Theme 4 - The learning environment can affect one’s decision to seek help	“There’s just a kind of atmosphere in medicine, everybody’s stressed, and you just get on with it. And I think a lot of doctors are stressed and overwhelmed; so I think you kind of just think it’s normal. I think it’s really sometimes you think, well, I might be struggling, but everybody else is; I’ve never felt that my psychological wellbeing is bad enough to seek professional help. So I think that makes you not want to seek help” P8, Y3
Themes 5 - Wellbeing services are perceived to be helpful once accessed, despite problems experienced	“When I hear about other students’ experiences when they get to a person [counselor], It’s really good, but sometimes getting that sort of right person can be a bit tricky… the counselors themselves are fantastic. They really do want to help, so great when you get through” P10, Y4
Theme 6 - Enhancing the accessibility of current medical school support and university wellbeing services	“I think there needs to be some work on the kind of reassure people that their competency and like fitness to practice won’t be questioned, because I think that’s a real big concern” P8, Y3

### Theme 1 - difficulty finding the wellbeing information prevents access to support

Medical students discussed various reasons hindering their access to wellbeing support or various obstacles they faced in the process. They claimed that the inability to find the correct information related to the appropriate wellbeing service when needed was the main barrier to receiving support.

“It was difficult to find that information, and I don’t think it should be difficult to find that information” P7, Y4.

### Theme 2 - long processes and waiting lists are deterring factors in help-seeking

Medical students faced challenges in finding information and navigating the processes of requesting wellbeing support, leading to potential overwhelm.

“I also feel like sometimes you have to jump through quite a few hoops to get to them” P7, Y4.

Another notable barrier highlighted was the extensive waiting list for service access. Medical students would seek help when their immediate situations would demand it, but university counseling services would often be available to offer support in a few weeks or months after the approach was made. This reputation of access problems seemed to deter medical students from seeking support since they knew that it would not be available to them in the near future.

“The waiting times can be pretty bad for the official university support counseling” P4, Y5.

### Theme 3 - confidentiality and fear of negative judgment are barriers to seeking help

Furthermore, many medical students believed that reaching out for help is a sign of weakness and a ‘black stain’ on their academic records. Medical students worried that their privacy could be breached, which could have a negative effect on their future careers. In addition to the large amount of pressure medical students faced already, concern about their fitness to practice pushed them further away from reaching out in dire situations.

“Fitness to practice was a big thing, so I was really worried that if I made it known that I needed kind of extra help, I was worried that I would kind of be questioned about my ability to be a doctor” P8, Y3.

### Theme 4 - the learning environment can affect one’s decision to seek help

Medical students reported facing relentless pressure to excel academically. Additionally, cultures that accept stress-related health problems as commonplace seemed to contribute to another barrier, preventing students from seeking help. This pervasive standard became toxic, particularly when medical students would internalize it as the norm and endure prolonged periods of stress.

“There’s just a kind of atmosphere in medicine, everybody’s stressed, and you just get on with it. And I think a lot of doctors are stressed and overwhelmed; so I think you kind of just think it’s normal. I think it’s really sometimes you think, well, I might be struggling, but everybody else is; I’ve never felt that my psychological wellbeing is bad enough to seek professional help. So I think that makes you not want to seek help” P8, Y3.

### Themes 5 - wellbeing services are perceived to be helpful once accessed, despite problems experienced

On a positive note, medical students seemed to appreciate a supportive environment when the staff was attentive and the support sources were actually accessed. When medical students managed to gain access to University counseling services, they reported a positive experience and found it significantly beneficial.

“When I hear about other students’ experiences when they get to a person [counselor], It’s really good, but sometimes getting that sort of right person can be a bit tricky… the counselors themselves are fantastic. They really do want to help, so great when you get through” P10, Y4.

In addition to seeking professional support, several students reported that strong family connections were a key source of support that helped alleviate stress. They found vocalizing their problems to someone close to them helpful in stress relief. In addition, the social interaction of seeking advice from peers gave them a new perspective and allowed them to better think through their problems before vocalising them.

“Probably the most useful thing I found with that was probably talking to others in the same situation. And I think that’s what made me feel the best speaking to other people who are also stressed and struggling, and then I didn’t feel like it was just me” P8, Y3.

Many students reported approaching a personal tutor for academic and pastoral support.

“I find it really useful to talk about [my stressors] with either my friends or my family, also my personal tutor as well” P23, Y5.

### Theme 6 - enhancing the accessibility of current medical school support and university wellbeing services

Medical students recommended improving the existing wellbeing support system to enhance accessibility and encourage seeking help for overall wellbeing. They suggested that services should be proactive and reach out to students rather than waiting for them to seek help. This approach was deemed important because individuals who would be truly depressed might not take care of themselves well; therefore, it was thought to be crucial for welfare services to actively identify and proactively offer them support to enhance their wellbeing.

“services should be proactive. So I think the service should reach out to students rather than wait for the students to come to them for the service. And this is for many reasons; one of them is that people who are truly depressed and were truly feeling very, very bad tend not to look after themselves very well, So you need to go out and try to fish them and define them and to offer them the support” P15, Y4.

Another crucial aspect that medical students brought up was the need for confidentiality when accessing support services. They recommended that medical schools explicitly circulate information on confidentiality measures to ensure that students feel comfortable reaching out to help.

“I think there needs to be some work on the kind of reassure people that their competency and like fitness to practice won’t be questioned, because I think that’s a real big concern” P8, Y3.

### Focus group results

#### Participant characteristics

For the focus groups, 22 participants completed the consent form and were included in the study. Participants were recruited in 7 focus groups, each group consisting of 3 to 4 in each group. Demographic information can be found in Table 1. Five themes and subthemes emerged from the focus group data; these highlighted detailed challenges related to accessing medical school support and university wellbeing services, offering additional insights beyond the interview findings. Additionally, suggestions were provided by medical students through reflective discussion around ways of enhancing the wellbeing support offered or to be offered to them, including implementation strategies. These are detailed in Table 3, which summarizes the outcomes of the focus group hybrid-thematic analysis.

**Table 3:** Focus group hybrid-thematic analysis outcomes

Themes	Sub-themes	Quotes
Theme 1 - Difficulty in identifying appropriate support due to lack of information or clarity regarding available resources/support		“ it might be a fact that it’s not well-published enough as to which services are specifically applicable to which circumstances” G4, P1
Theme 2 - The complexity of procedures and the extended period of time acted as deterrents for students seeking help, discouraging them from seeking support	Subtheme 2a - Confusing processes for requesting wellbeing support, and unclear directions, can further exacerbate the negative impact of long waiting times	“ counseling at the moment, the waiting lists are months long and as a medical student, especially in your clinical years, the counseling times from the university and the appointments available to you from the university ones are very inconvenient” G1, P3
Theme 3 - The pervasive culture within medical schools can significantly impede students from seeking help	Subtheme 3a - Normalizing poor mental health prevented students from seeking help	“ I don’t think it’s fair to normalizing that; I think it is very normal for doctors to do that because they’re all dealing with trauma; and I think the culture is to just deal with it and not seek support and kind of bury it, and in many doctors, I talked to, that is the case. I think that’s kind of a cultural problem rather than an individual problem, but obviously, that’s not the easiest thing to change” G2, P3
	Subtheme 3b - Self-beliefs and feeling sceptical about the usefulness of the services undermined help-seeking	“ It is about medical students being independent, but sometimes they’re expected to be independent as well. Not just with studying but also with looking after themselves” G1, P3
	Subtheme 3c - Confidentiality issues impeded students from seeking help	“ I think confidentiality probably is a real thing, I think we want reassurance from the very beginning that it is completely confidential and it is the appropriate thing to seek support” G7, P2
	Subtheme 3d - Fear of stigma acted as a deterrent to asking for help	“ I still think that some of it may be because there’s still a stigma attached to seeking services; sometimes I think that stigma that comes around seeking services may put people off as well” G2, P1
Theme 4 - Improving the structure and format of the current medical school support and University wellbeing service to make them more accessible	Subtheme 4a - Providing clear information and regular intervals of support can help in choosing the right source of support	“ To have like a diagram to show like what actually happens, and what would happen if you need this sort of help or that’s helps” G1, P1
	Subtheme 4b - Making the services proactive and accessible within a reasonable timeframe to foster better wellbeing provision	“ I believe it would be beneficial to have someone available to speak with individuals in crisis, such as by sending a referral or reaching out to those on the waiting list. These check-ins wouldn’t be full sessions but rather brief 15-minute interactions” G7, P2
	Subtheme 4c- Improving the safety net by acknowledging students’ stressors in the medical school environment and providing reassurance around confidentiality	“ emphasize that it’s confidential. It won’t affect your study. Like it won’t go on your record and these people are professionally trained to help you, which makes a huge difference” G6, P1
Theme 5 - Student responsibility for maintaining positive wellbeing	Subtheme 5a- Self-care through hobbies and personal time promote good mental health	“if we are taught better to recognise these signs of impending crisis in ourselves and others, then we can better uplift each other because we can turn to our friends and say, Hey, I’m really worried about you, because I’m seeing this, this and this, and that makes me concerned. The other thing I would add is I think it’s very important to put that, we should do our absolute best to support one another and be kind to one another and uplift one another’’ G1, P4

### Theme 1 - Difficulty in identifying appropriate support due to lack of information or clarity regarding available resources/support

One major challenge highlighted by medical students pertained to the difficulty they face in identifying appropriate support, a challenge that existed even before COVID-19. They expressed frustration due to a lack of clarity regarding the available resources, which hindered their ability to seek the help they needed. Specifically, they mentioned that there seemed to be a lack of effective dissemination or communication regarding the specific services that are applicable to different circumstances. They expressed concerns about the clarity and accessibility of information regarding which services would be most relevant and suitable for their particular situations.

“ it might be a fact that it’s not well-published enough as to which services are specifically applicable to which circumstances” G4, P1.

Additionally, the lack of clarity was further compounded by students’ varying levels of awareness, where some services would be well-known while others remain unfamiliar. As a result, accessing the necessary support becomes a challenging task for these students.

 “ Medical students specifically contact the Counseling Service and don’t know we have an actual specific counselor for the medical students, and it’s not sort of published anywhere very well’’ G4, P1.

This theme is substantiated by a priori codes aligned with previous interview findings, denoting the lack of clarity around the available wellbeing support.

### Theme 2 - the complexity of procedures and the extended period of time acted as deterrents for students seeking help, discouraging them from seeking support

#### Sub-theme 2a - confusing processes for requesting wellbeing support and unclear directions can further exacerbate the negative impact of long waiting times

Medical students highlighted that the university’s counseling service was often slow to respond to emails and requests for appointments. Additionally, they reported long waiting lists for counseling services, with wait times stretching to months. These delays in accessing support not only exacerbated the students’ distress but also impeded their ability to address their mental health needs promptly. The challenges became even more pronounced during the COVID-19 pandemic when the mental health of many individuals, including medical students, was under increased strain. Additionally, due to the demanding schedules, especially during their clinical years, medical students often struggled to find time to attend appointments without compromising their studies. The clash between their busy schedules and the limited availability of counseling sessions made it challenging for them to prioritize their wellbeing.

“ counseling at the moment, the waiting lists are months long and as a medical student, especially in your clinical years, the counseling times from the university and the appointments available to you from the university ones are very inconvenient” G1, P3.

These align with previous findings from the interviews, confirming the theme through a priori and posterior codes.

### Theme 3 - the pervasive culture within medical schools can significantly impede students from seeking help

#### Sub-theme 3a - normalizing poor mental health prevented students from seeking help

Across all groups, students discussed the detrimental effects of normalizing poor mental health within the medical profession, which hindered their willingness to seek help. They described how they would sometimes suffer in silence, trying to fit in with the demanding culture, which could harm their psychological wellbeing. Because they acknowledge that being stressed and overworked is a qualifying factor, they did not believe that their problems were bad enough to warrant assistance. In particular, they noted that dealing with trauma was considered to be ‘part of the job’, and that the culture would often discourage them from seeking assistance or discussing personal challenges openly. Many students shared the feeling that ‘burying’ these experiences was the expected approach. Although they also recognized that changing this cultural norm would not be easy, they nonetheless acknowledged the need for a shift to support their mental health and wellbeing better.

“ I don’t think it’s fair to normalizing that; I think it is very normal for doctors to do that because they’re all dealing with trauma; and I think the culture is to just deal with it and not seek support and kind of bury it, and in many doctors, I talked to, that is the case. I think that’s kind of a cultural problem rather than an individual problem, but obviously, that’s not the easiest thing to change” G2, P3.

#### Sub-theme 3b - self-beliefs and feeling skeptical about the usefulness of the services undermined help-seeking

Students’ self-beliefs and skepticism about the usefulness of available support services significantly hindered help-seeking behavior. The competitive nature of medical school was thought to foster a sense of self-reliance, where students might hesitate to ask for help, fearing it could be perceived as a sign of weakness or incompetence. While some a priori codes supported this subtheme, it primarily emerged from the analysis during the research process (a posteriori codes).

“ It is about medical students being independent, but sometimes they’re expected to be independent as well. Not just with studying but also with looking after themselves” G1, P3.

#### Sub-theme 3c - confidentiality issues impeded students from seeking-help

Confidentiality issues within medical school settings could also act as a barrier to seeking help. Students tended to worry about the privacy and confidentiality of their personal struggles, fearing that seeking assistance could negatively affect their academic progress or future careers. This fear of breaching confidentiality created a sense of vulnerability and reluctance to disclose their challenges, further deterring them from seeking the help they needed.

“ I think confidentiality probably is a real thing, I think we want reassurance from the very beginning that it is completely confidential and it is the appropriate thing to seek support” G7, P2.

#### Sub-theme 3d - fear of stigma acted as a deterrent to asking for help

The fear of being labelled as mentally unstable or weak was found to be profoundly impacting students’ willingness to ask for help. The drive to appear resilient and invulnerable could lead students to suppress their struggles, inadvertently perpetuating a cycle of silent suffering. The fear of being stigmatized within their peer group and professional community became a powerful deterrent to seeking the necessary support.

“ I still think that some of it may be because there’s still a stigma attached to seeking services; sometimes I think that stigma that comes around seeking services may put people off as well” G2, P1.

### Theme 4 - improving the structure and format of the current medical school support and University wellbeing service to make them more accessible

#### Sub-theme 4a - providing clear information and regular intervals of support can help in choosing the right source of support

The focus group findings highlighted the necessity of improving wellbeing services to cater for medical students’ needs. Students reaffirmed previous suggestions (a priori codes) and provided detailed explanations for implementing these suggestions effectively. Additionally, they have put forth additional recommendations (posteriori codes) for enhancing academic and pastoral support and other wellness services within medical schools.

The findings from the focus group discussions confirmed the need to enhance the effectiveness of advertising the available wellbeing support services as discussed in the interviews (priori codes). Students recommended several further strategies, including regular verbal reminders about the services.

“ a verbal reminder as well as the emails, maybe that could be something to change” G7, P3.

They also proposed the creation of a comprehensive diagram that would outline the various support options available, accompanied by clear steps for accessing them.

“ To have like a diagram to show like what actually happens, and what would happen if you need this sort of help or that’s helps” G1, P1.

Additionally, students expressed a desire for a designated person within the medical school’s wellbeing department who would serve as a well-known initial point of contact, capable of promptly directing students to the appropriate resources.

“ Having the one person in the wellbeing department in the med school who everyone is made aware that they could go to and then that person know where to then send you just so having it more streamlined and being able to be sent to the right place immediately to like access the support that you need” G4, P2.

Furthermore, they expressed the need for a centralised resource that would provides clear information on whom to contact based on the specific type of support required, distinguishing between academic and wellbeing support.

“ I want to already know that there is a place that I can go, and it’s going to tell me who I can contact based on the kind of support we need where it says if you need academic support, contact these people, while if you need support with your wellbeing, contact these people’’ G6, P3.

#### Sub-theme 4b - making the services proactive and accessible within a reasonable timeframe to foster better wellbeing provision

Students provided detailed explanations on how the services could become proactive in addressing the identified needs. They emphasized the significance of allocating sufficient resources, including increased numbers of designated welfare staff, to meet the growing demand for mental health services and reduce waiting lists. Specifically, their proposal involved the appointment of welfare staff who could work flexible hours, including evenings and early mornings, to ensure accessibility for individuals in various crises or in need. These staff members would need to be reachable through phone, email, chat box messaging, and video calls, creating a broad and easily accessible service for individuals.

“ Employing more staff to work more flexible hours, extending into the night and early morning and who are accessible over the phone, email, chat box messaging and video call, to make the service as broad and easy to reach as possible for people in all kinds of crisis and need” G3, P3.

Recognizing the urgency of the current mental health crisis, the students emphasized the importance of providing timely assistance and support. In line with this, they proposed conducting brief check-in sessions for students on waiting lists, lasting around 15 min. These sessions would assess progress and offer additional support to those awaiting services.

“ I believe it would be beneficial to have someone available to speak with individuals in crisis, such as by sending a referral or reaching out to those on the waiting list. These check-ins wouldn’t be full sessions but rather brief 15-minute interactions” G7, P2.

#### Sub-theme 4c - improving the safety net by acknowledging students’ stressors in the medical school environment and providing reassurance around confidentiality

To enhance the safety net for students in medical schools, students recommended fostering an atmosphere that reduces stigma and normalizes medical students’s mental health states whilst going through stressful periods. This message was thought to be crucial for promoting student wellbeing and encouraging the utilization of support services. In line with this, students suggested implementing workshops, seminars, or orientation programs to openly address stressors and provide strategies for managing them.

“ making issues of wellbeing sort of discussed more openly at the beginning of medical school Lecture Series as part of the seminars workshops that we have, would help” G1, P3.

Providing clear and early reassurance regarding the confidentiality of wellbeing services was also deemed crucial. By emphasizing that seeking help would not adversely affect academic records and ensuring students that their privacy would be protected, they could be made to feel more at ease and motivated to use the available resources. This initial reassurance would be vital in creating a supportive environment where students feel comfortable seeking help without fear of negative consequences.

“ Emphasize that it’s confidential. It won’t affect your study. Like it won’t go on your record and these people are professionally trained to help you, which makes a huge difference” G6, P1.

Additionally, students suggested organizing social events to create a friendly and sociable environment in medical schools. These events would serve as reminders of the peer support network available to students and emphasize the significance of understanding and empathy from faculty and staff. By fostering a sense of community through social gatherings, medical schools could strengthen the support system for students and promote an environment where understanding and empathy would be valued.

“ I think maybe they even have some social events a few times a year, just to you know, make it feel like the med schools are more friendly, sociable places to remind people about all the peers you have around you that you can talk to. I think that would be something really valuable” G6, P1.

### Theme 5 - student responsibility for maintaining positive wellbeing through community, connection and self-care

#### Sub-theme 5a - self-care through hobbies and personal time promotes good mental health

This emergent theme and subthemes, identified through mainly posterior coding, underscored the students’ acknowledgement of their role in promoting wellbeing. In the focus groups, students engaged in meaningful discussions about their own responsibility for their wellbeing. They acknowledged the importance of self-awareness and recognizing signs of distress in themselves. They emphasized the need for open and honest communication, both with oneself and with others, to address concerns and seek support.

 “ if we are taught better to recognize these signs of impending crisis in ourselves and others, then we can better uplift each other because we can turn to our friends and say, Hey, I’m really worried about you, because I’m seeing this, this and this, and that makes me concerned. The other thing I would add is I think it’s very important to put that, we should do our absolute best to support one another and be kind to one another and uplift one another’’ G1, P4.

Students also emphasized the significance of self-care practices, such as engaging in activities they would enjoy, maintaining a balanced lifestyle, and prioritizing their mental and physical health.

“ having hobbies and having time off and spending time with friends and family’’ G2, P3.

Please refer to Table 4 for a succinct summary of the medical students’recommendations for enhancing wellbeing support.

**Table 4:** Recommendations for enhancing wellbeing support

Aspect	Recommendations for Enhancing Wellbeing Support
Providing Clear Information and Timely Support	- Implementation of strategies like verbal reminders and visual diagrams outlining support options - Designation of a well-known initial point of contact within the medical school’s wellbeing department - Prompt direction of students to appropriate resources
Proactive Welfare Measures and Accessibility	- Increased staffing to meet growing demand for mental health services and reduce waiting lists - Appointment of welfare staff available through various communication channels and flexible hours - Simplification of administrative procedures for accessing support services - Conducting brief check-in sessions for students on waiting lists
Strengthening the Safety Net	- Promotion of a culture valuing seeking help as a sign of strength - Implementation of workshops or seminars addressing stressors openly and providing management strategies - Clear reassurance around confidentiality - Offering confidential support options to alleviate concerns about professional image - Organizing social events to foster a friendly and sociable environment
Fostering Better Communication	- Improved communication among all stakeholders in the medical school - Establishing solid relationships among education staff - Transparency, centralization of information, and clear instructions for accessing the wellbeing support services
Enhancing Consistency in Support Services	- Ensure access to support personnel and maintain consistent engagement - Initiate proactive communication from support personnel, particularly during challenging periods - Clear and accessible support services with greater clarity regarding availability
Student Responsibility for Maintaining Positive Wellbeing through Community, Connectedness, and Self-Care	- Prioritizing supportive relationships, meaningful connections - Self-Care strategies: Role of self-awareness, open communication, and engaging in enjoyable activities for mental and physical health - Fostering a supportive community and promoting inclusivity to achieve a healthy balance between academics and personal life

### Discussion and integration of findings

#### Barriers to accessing medical school support and university wellbeing services can prevent help-seeking

The interview part of the study uncovered significant barriers hindering medical students from seeking help, including difficulties in accessing wellbeing information, lengthy processes, the challenging learning environment, confidentiality concerns, and fear of judgment. A follow-up series of focus groups reinforced these barriers, offering deeper insights into their impact on students’ accessing support services and their overall wellbeing. Students suggested improvements in the accessibility, presentation format and structure or delivery of support services, emphasizing the need to address these challenges for better student wellbeing.

Medical students in both the interview and focus group data have pinpointed various potential sources of academic and wellbeing support, including personal tutors, academic and administrative staff at medical schools, placement supervisors, and wellbeing support services. However, previous research findings have indicated that several medical students have perceived inadequate resources allocated to their mental health [17, 35]. The findings of the present study revealed that most medical students would have some knowledge of the existing wellbeing services, but this knowledge would not be complete. Both the interview and focus group data highlighted the lack of clarity and extended waiting times as common concerns. This lack of clarity prevented students from confidently accessing services when needed, and lengthy waiting lists discouraged help-seeking, especially in urgent situations. These findings are consistent with research conducted in various countries, including the UK, Australia, and the United States [16, 36, 37]. This pattern of findings suggests that the challenges in accessing medical school support and university wellbeing services are not unique to a particular region but represent a widespread concern among students in diverse educational settings and geographical contexts. These challenges have persisted both before and during the COVID-19 pandemic. Prior research has also underlined the persistent nature of these difficulties, which have worsened amidst the pandemic’s challenges [14]. This is concerning since medical students have previously reported needing help making time to seek help, and the added delay might discourage them from reaching out in times of need.

The pervasive stigma surrounding mental health seems to have led some medical students to refrain from seeking help for their mental health issues, even though there seemed to be a widespread emphasis on promoting wellbeing through learning resources and support services. In line with previous studies [38], many medical students tended to believe that seeking support is a sign of weakness and will leave a mark on their record and deem them unfit to become a doctor. The results of this study lend credence to the idea that medical students’ views on mental health can be shaped and reinforced by the environment where faults are hidden, poor mental health is normalized, and illness is a sign of weakness and failure. Like our study results, previous research has found that medical students in the UK [15, 38] experience fear of stigma when discussing issues about their wellbeing. To conform to the pressures of medical culture, medical students reported often struggling in silence, eventually damaging their mental health. Since they would recognize that being stressed and overworked is a ‘qualifier’, they would not consider their conditions severe enough to request help. This is consistent with a similar report from students at a US university, who described this sense of normality as a deterrent to seeking help [39]. Universities need to reinforce a climate whereby asking for help would be considered to be an opportunity for growth instead of a sign of weakness. All students should feel comfortable and be able to access support services regardless of their academic standing. Through encouraging conversations around mental health and wellbeing, staff in various welfare roles can also enact their responsibility of breaking down the stigma surrounding these topics and in ensuring consistency in support provision. Previous research has highlighted the significance of medical schools offering a supportive environment for their students through continuity of care [17].

#### Suggestions for improving wellbeing support to meet medical students’ needs (pastoral - academic)

Analysis of the data revealed that students did not wish to have new resources or interventions/services provided to them, but instead, they wanted the existing support services to be improved to better suit their needs. In response to these concerns, students offered a range of suggestions for enhancing the accessibility of current support services within the medical school and university settings. In their efforts to boost accessibility, students provided valuable recommendations for refining the university’s wellbeing support services to better align with their needs. These findings are consistent with previous studies, which have emphasized the significance of clear communication [40], reduced stigma [38], and efficient service provision in supporting students’ mental health and wellbeing [37].

In the discussions, students have provided thorough explanations to outline their perceived necessary changes and additional support required for the improvement of the existing sources of wellbeing. Moreover, they have proposed further recommendations to enhance pastoral and academic support, along with other wellbeing services within medical schools. Their main focus was on the need to improve the regularity and clarity of the service information provided. Medical students struggled with various personal or academic problems; hence, they needed to be informed of the services that would best suit them. Students suggested one way to promote access to the services was to clarify the information around the person or the services they should approach concerning their specific situations. Whether a student would prefer support from within or outside the University also varies; therefore, details of both institutional and external options would need to be presented clearly. Prior research has found that wellbeing support services were limited for second-year students due to inadequate promotion of available resources [41]. To enhance access, it was recommended to advertise wellbeing services to all year groups clearly and concisely.

Furthermore, multiple studies have emphasized the significance of positive relationships, effective communication, structured schedules, and timely access to services for students’ wellbeing [17, 18, 37]. Our data support these findings, advocating for brief check-ins for waitlisted students, prioritizing immediate needs, and implementing workshops to openly address stressors. Confidentiality and safety net enhancement were deemed vital for a secure environment, aligning with strategies adopted in Australia and New Zealand [42]; the authors of the study suggested that medical schools could play an essential role in reducing the stigma associated with mental health issues, fostering students’ ability to recognize when they needed help, and facilitating their access to professional care, which is in line with the findings from our study.

It’s worth noting that students recognized the critical role of prioritizing their own wellbeing, which is consistent with research that underscores the value of self-care practices and nurturing supportive connections in maintaining positive mental health among students [43]. The students emphasized the need for self-awareness, open communication, and seeking appropriate support when needed. Engaging in hobbies, personal time, and prioritizing mental and physical health were also highlighted as essential self-care practices [42]. Building strong personal relationships and creating a sense of belonging have been shown to enhance mental health and wellbeing among medical students [6].

It is interesting to note that medical students seemed to value a supportive environment where they would find encouraging and caring staff members and approachable support sources. Where medical students have persevered through the system, they report positive experiences, noting and appreciating the convenience of the services. However, these students hoped that the support sources would become even more approachable, potentially further enhancing their help-seeking and promoting wellbeing. It is evident that some students sought help from personal and professional sources, but others faced barriers to accessing such support. Medical schools need to consider how they can best provide a supportive and nurturing environment for students and offer tailored resources to help them cope with the challenges and stress of medical training.

Medical training presents students with unique challenges, and it is essential that medical institutions prioritize the mental health and wellbeing of their students. Improving access to support services by improving their structure and format and taking a comprehensive approach to psychological support provision could help promote medical students’ mental health and wellbeing [42]. Individual behaviors and environmental influences, as well as relationships among teachers, students, professional services, and the greater community, should all be considered by medical schools. Studies have affirmed the positive impact of wellbeing programs and interventions, such as mindfulness and stress reduction [20, 44]. Integrating physical activity into the curriculum has also been demonstrated to improve medical students’ wellbeing [9]. These approaches have been thought to assist students in balancing the demands of their studies and clinical work, eventually enhancing their capacity to provide patients with high-quality care. Ensuring students can openly share mental health concerns and receive continuous care in a supportive environment is crucial. This would also foster cultural competencies, enhancing their ability to serve diverse populations throughout their studies and beyond [10].

This qualitative study has yielded practical implications by uncovering the primary barriers that seem to hinder students’ access to mental health support services. It offers student-driven recommendations tailored to the given barriers encountered by students, along with advanced strategies to enhance access to such support. In essence, it could be argued that this study contributes to the broader discourse on student wellbeing promotion in educational institutions by addressing the specific challenges faced by medical students and offering targeted solutions from students’ perspectives. It reinforces the idea that effective support systems would be informed by student preferences and implemented through collaborative efforts between students and the institution.

#### Study strengths and limitations

The study presents several notable strengths. First, it has conducted a comprehensive exploration of the barriers that medical students reported to encounter when seeking access to support services within the medical schools and the university context. Combining interviews and focus group discussions, the research has delved deeply into the topic, considering the perspectives of those directly affected and seeking their practical recommendations for improving the accessibility and effectiveness of support services for medical students irrespective of where they are based.

Despite its strengths, the authors have acknowledged several limitations that warrant consideration. The study findings have primarily focused on the University of Nottingham; as such, the generalisability of these findings to other medical schools in the UK or other countries with potentially different institutional structures and cultural contexts may be limited. A further limitation of the study is that there may be a difference in the willingness of students to share their experiences, resulting in a somewhat incomplete picture of the barriers that were reported to be faced by medical students. Other potential factors that could possibly be contributing to the barriers to medical students’ access to support provision, such as family circumstances and cultural or religious aspects, might have been neglected in this study, given that they were not emphasized by the participants in the interview or focus group discussions.

### Future research

Building on the present study findings, future research could adopt a longitudinal study design to monitor evolving challenges, pilot interventions to test out recommendations and explore cultural differences in support needs. Collaborative efforts between universities and healthcare institutions would also be worth pursuing in an effort to provide a broader lens into the trajectory of challenges and support needs across the healthcare continuum.

## Conclusion

Given that medical students reportedly strive to become efficient future healthcare professionals, it is imperative to acknowledge and address the barriers hindering their access to wellbeing support services when there is a perceived need for these. This study emphasizes the need for destigmatizing mental health, fostering supportive relationships, and providing consistent and tailored support services to medical students to encourage their help-seeking attitudes and behavior. Implementing the practical recommendations that emerged from this study, where applicable, could potentially enhance support systems within medical schools and universities.

## Data Availability

The datasets analyzed in this review are available upon request. Please contact the corresponding author

## Abbreviations

COVID-19: Coronavirus disease 19
NVivo: Navigating viewpoints, images and value observed
